# Supplementary material for: Transcriptomic Analysis Reveals Intrinsic Abnormalities in Endometrial Polyps
Source: Int J Mol Sci. 2024 Feb 22;25(5):2557. doi: 10.3390/ijms25052557 (PMC10932376; doi:10.3390/ijms25052557)
Supplement: Supplementary file 1 [file ijms-25-02557-s001.zip › ijms-2878871-supplementary.pdf]

Supplementary Materials to

Transcriptomic Analysis Reveals Intrinsic Abnormalities in Endometrial Polyps

Supplemental Figures

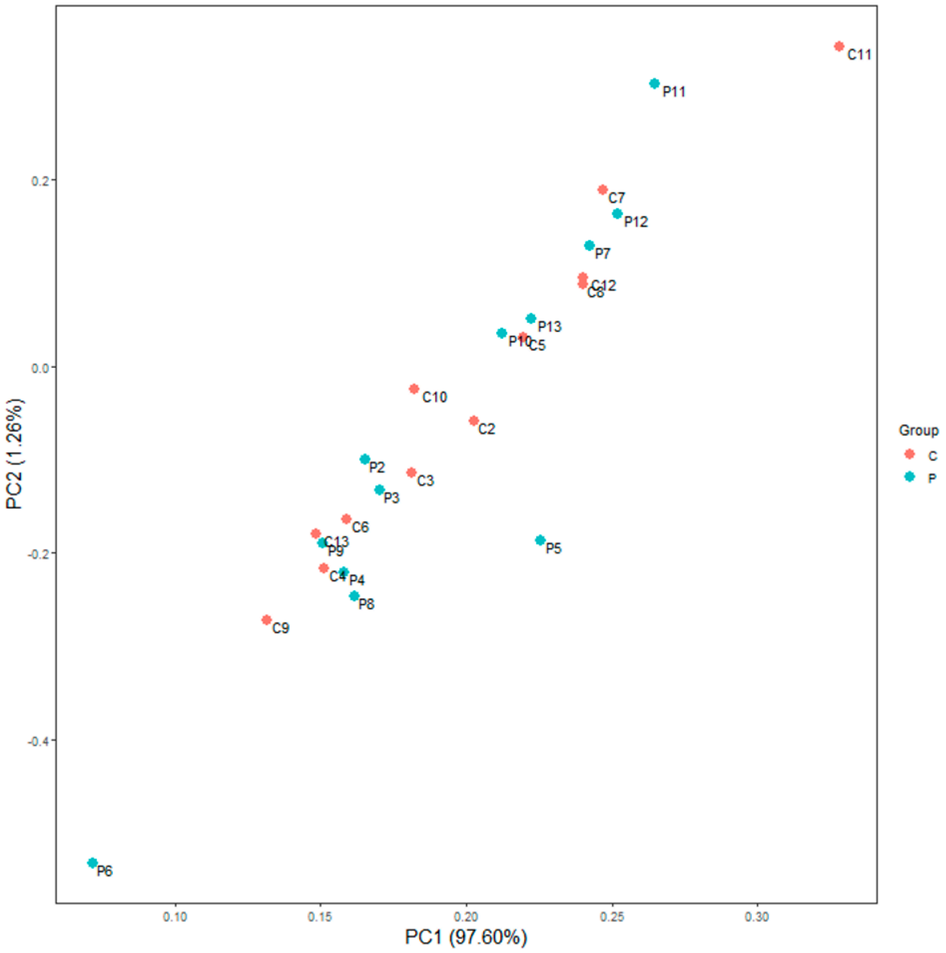

Figure S1. PCA analysis of the RNA-seq dataset presented in this study used the prcomp function in R packages. This figure has been redrawn and shown in Figure 1a.

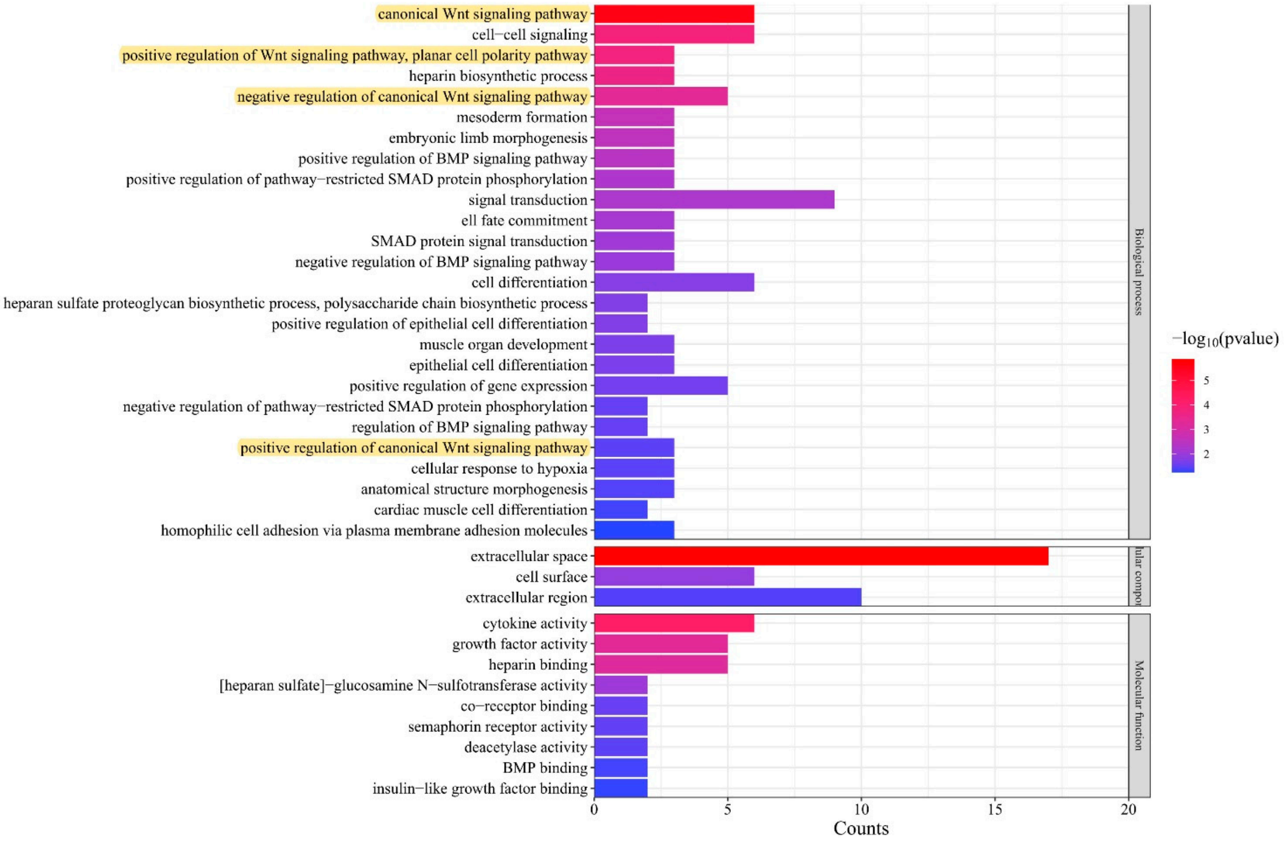

Figure S2. GO enrichment analysis for the DEGs in the PPI subcluster one.

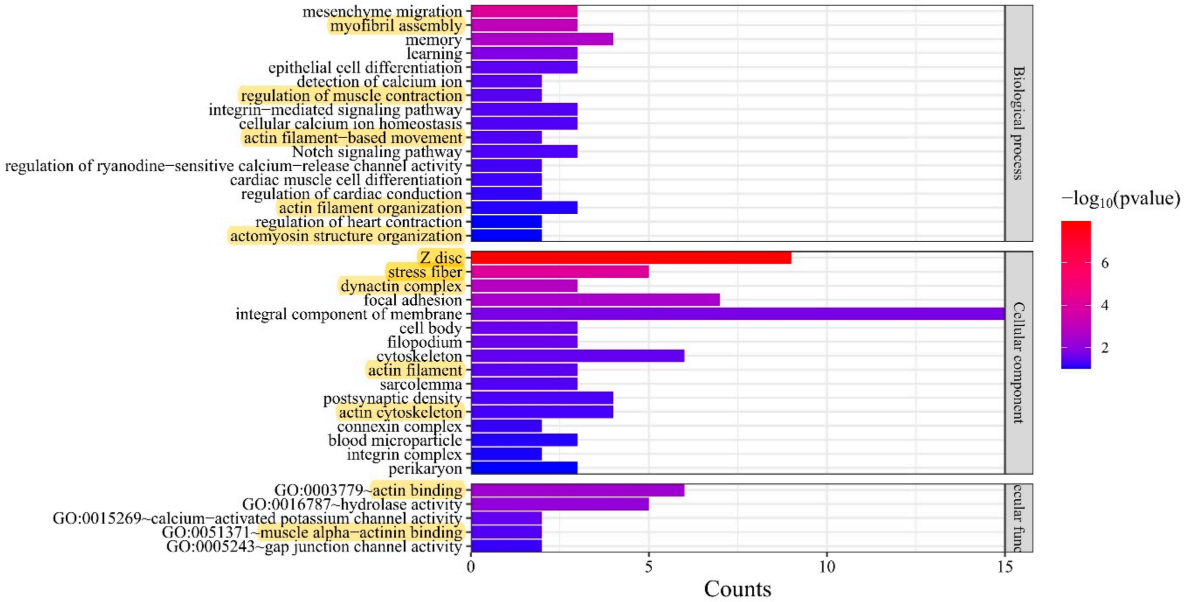

Figure S3. GO enrichment analysis for the DEGs in the PPI subcluster two.

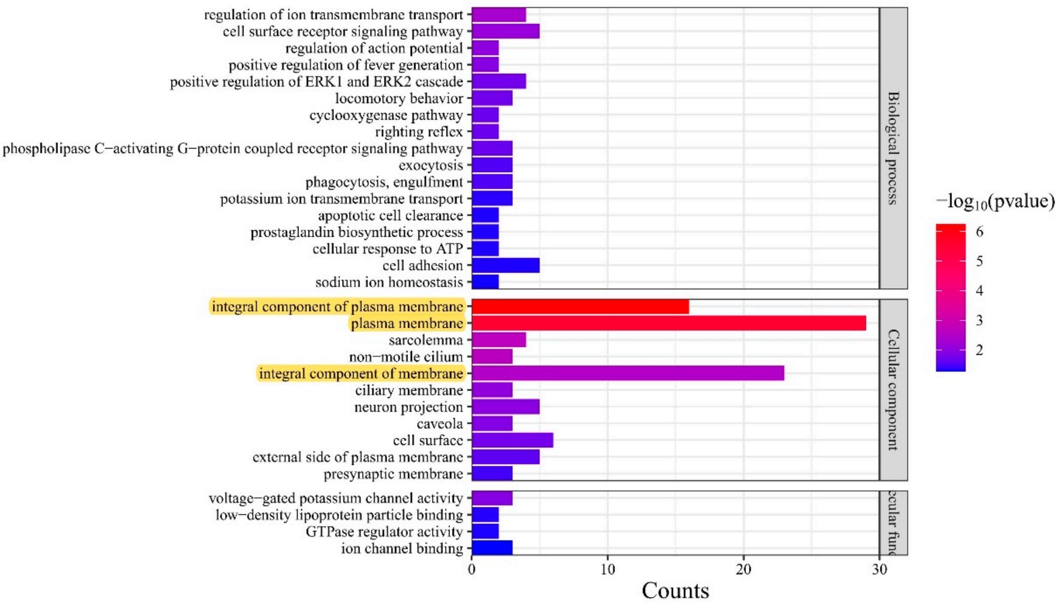

Figure S4. GO enrichment analysis for the DEGs in the PPI subcluster three.

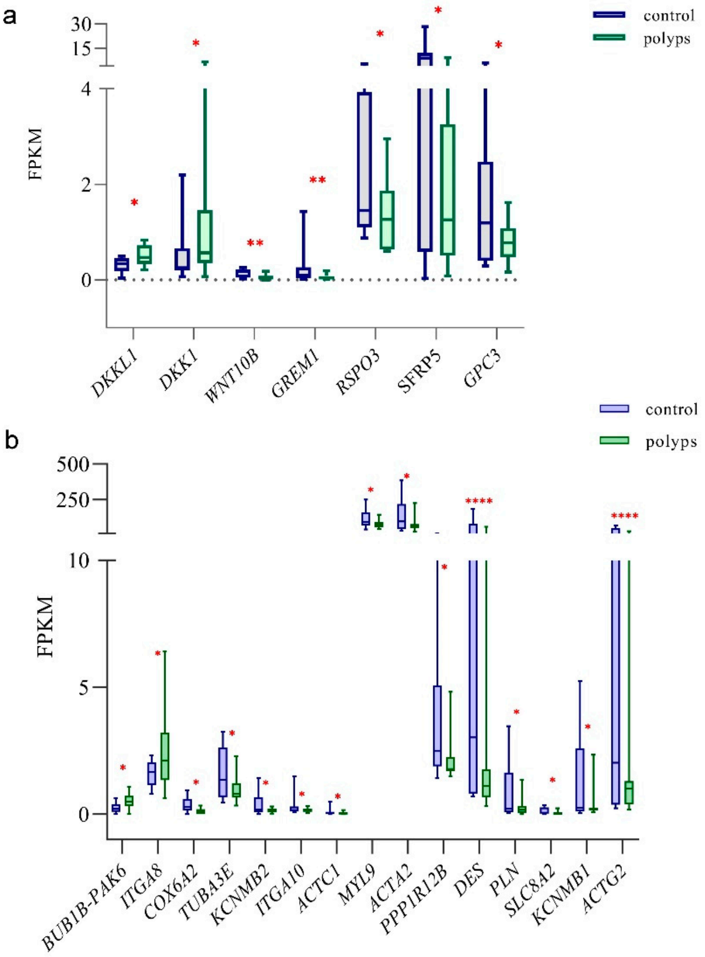

Figure S5. The boxplot of Wnt signaling pathway (a) and muscle function-related genes (b). * $p < 0.05$, ** $p < 0.01$, , **** $p < 0.0001$.

Supplemental Tables

Table S1. Physiological parameters of the subject.

No.	Age	BMI	Polyp size	Endometrial thickness	OP day	Cycle interval	AMH
1	41	19.30	0.7*0.4cm	1.15	9	25-29	
2	41	20.03	0.52cm	1.2	9	25-26	1.94
3	39	19.70	no data	0.82	10	28-30	2.65
4	43	24.10	no data	0.8	10	26-28	0.23
5	37	19.00	0.5cm, 0.2cm	0.83	12	40-45	12.19
6	37	24.30	no data	0.72	9	45-60	6.09
7	39	24.56	0.3*0.1cm	1.02	14	50-60	5.40
8	40	21.80	0.6*0.4cm, 0.4*0.4cm	1.4	11	20-25	2.75
9	37	–	0.8*0.4cm	not available	7	28-30	3.52
10	39	21.50	no data	0.71	13	26-28	3.85
11	40	19.70	0.7cm	0.9	9	28-30	3.69
12	36	20.40	0.7*0.4cm	0.95	7	25-28	3.68

OP: operation

Table S2. Comparison of gene expression levels between polyp tissue and corresponding adjacent endometrium.

Symbol	Gene description	Gene ID	Fold change - P/C*	*p*-Value
**Up-regulated genes**				
AC112503.1	-	ENSG00000272678	17.0090	0.0332
AC024084.1	-	ENSG00000230649	12.0701	0.0211
MYT1L	myelin transcription factor 1 like	ENSG00000186487	10.2414	0.0134
AL031283.1	-	ENSG00000228140	10.1607	0.0356
NDST3	N-deacetylase and N-sulfotransferase 3	ENSG00000164100	7.0427	0.0129
AC097375.1	-	ENSG00000248991	6.6300	0.0314
NDST4	N-deacetylase and N-sulfotransferase 4	ENSG00000138653	6.4560	0.0141
AC018845.1	-	ENSG00000259821	6.4537	0.0497
CLC	Charcot-Leyden crystal galectin	ENSG00000105205	5.4192	0.0212
HGC6.3	-	ENSG00000235994	5.2353	0.0469
BX470102.1	-	ENSG00000238279	5.0843	0.0357
AADACL2-AS1	-	ENSG00000242908	4.6991	0.0286
HOXA13	homeobox A13	ENSG00000106031	4.5808	0.0006
FAM166A	family with sequence similarity 166 member A	ENSG00000188163	4.1297	0.0457
GJC3	gap junction protein gamma 3	ENSG00000176402	3.9476	0.0388
AL049650.1	novel transcript	ENSG00000256566	3.7906	0.0039
KRT5	keratin 5	ENSG00000186081	3.7371	0.0134
PCDH15	protocadherin related 15	ENSG00000150275	3.6222	0.0333
AC068831.1	-	ENSG00000258384	3.6177	0.0429
AC097658.1	-	ENSG00000228981	3.5458	0.0484
AL358075.2	-	ENSG00000227857	3.5039	0.0336
MARCO	macrophage receptor with collagenous structure	ENSG00000019169	3.4373	0.0082
AC004080.2	-	ENSG00000253508	3.3061	0.0433
AL139005.1	-	ENSG00000279393	3.2746	0.0340
AC073349.1	-	ENSG00000189316	3.0636	0.0362
AL109811.3	-	ENSG00000277726	3.0141	0.0485
TMCO1-AS1	-	ENSG00000224358	2.9514	0.0071
AC005256.1	-	ENSG00000267073	2.9241	0.0335
MT-TM	mitochondrially encoded tRNA-Met (AUA/G)	ENSG00000210112	2.8766	0.0260
LINC01747	-	ENSG00000230400	2.8689	0.0155
AL034374.1	-	ENSG00000271367	2.6840	0.0432
C4orf50	chromosome 4 open reading frame 50	ENSG00000181215	2.6242	0.0243
SYPL1P2	-	ENSG00000265683	2.6093	0.0015
CORIN	corin, serine peptidase	ENSG00000145244	2.5524	0.0115
AC011479.4	-	ENSG00000286037	2.4865	0.0007
AC074286.1	-	ENSG00000236501	2.4609	0.0370
LRRC37A9P	-	ENSG00000271013	2.4432	0.0082
DKK1	dickkopf WNT signaling pathway inhibitor 1	ENSG00000107984	2.4271	0.0239
PYY2	peptide YY 2 (pseudogene)	ENSG00000237575	2.3960	0.0350
TCAF2P1	TRPM8 channel associated factor 2 pseudogene 1	ENSG00000159860	2.3908	0.0473

AC107982.3	-	ENSG00000265478	2.3839	0.0257
AL034430.2	-	ENSG00000285723	2.3408	0.0201
AC112206.2	-	ENSG00000249364	2.2848	0.0342
CEP164P1	centrosomal protein 164 pseudogene 1	ENSG00000226937	2.1907	0.0273
KRT36	keratin 36	ENSG00000126337	2.1672	0.0366
AF064860.2	-	ENSG00000231713	2.1630	0.0059
AC079848.1	-	ENSG00000250012	2.1031	0.0254
AC093635.1	-	ENSG00000272663	2.0967	0.0356
IDH1-AS1	IDH1 antisense RNA 1	ENSG00000231908	2.0888	0.0160
AL355353.1	-	ENSG00000270761	2.0813	0.0007
CCN5	cellular communication network factor 5	ENSG00000064205	2.0692	0.0336
ELAVL2	ELAV like RNA binding protein 2	ENSG00000107105	2.0205	0.0482
NELL2	neural EGFL like 2	ENSG00000184613	2.0096	0.0391
AL080250.1	-	ENSG00000225793	2.0024	0.0133
AC079250.1	-	ENSG00000230979	1.9638	0.0046
BUB1B-PAK6	BUB1B-PAK6 readthrough	ENSG00000259288	1.9489	0.0287
ATP13A4	ATPase 13A4	ENSG00000127249	1.9393	0.0254
RPL10P19	-	ENSG00000233609	1.9366	0.0498
CXCL14	C-X-C motif chemokine ligand 14	ENSG00000145824	1.9340	0.0477
AL138752.2	-	ENSG00000255872	1.9100	0.0421
LINC00345	long intergenic non-protein coding RNA 345	ENSG00000235660	1.8788	0.0100
AC138150.2	-	ENSG00000267288	1.8650	0.0415
AL513190.1	-	ENSG00000278831	1.8511	0.0460
AC010980.2	-	ENSG00000267034	1.8326	0.0371
AC099522.2	-	ENSG00000272525	1.8168	0.0422
IL13RA2	interleukin 13 receptor subunit alpha 2	ENSG00000123496	1.7976	0.0445
GAS5-AS1	GAS5 antisense RNA 1	ENSG00000270084	1.7860	0.0101
AL137127.1	-	ENSG00000272084	1.7763	0.0474
BMP7	bone morphogenetic protein 7	ENSG00000101144	1.7687	0.0178
SH3GL2	SH3 domain containing GRB2 like 2, endophilin A1	ENSG00000107295	1.7380	0.0480
AC090587.2	-	ENSG00000229368	1.7056	0.0225
LNCOC1	-	ENSG00000253741	1.6878	0.0245
AL445472.1	-	ENSG00000227486	1.6730	0.0024
IGFBPL1	insulin like growth factor binding protein like 1	ENSG00000137142	1.6536	0.0476
SLITRK5	SLIT and NTRK like family member 5	ENSG00000165300	1.6531	0.0036
TMEM71	transmembrane protein 71	ENSG00000165071	1.6330	0.0130
XKR4	XK related 4	ENSG00000206579	1.6104	0.0194
MMP23B	matrix metallopeptidase 23B	ENSG00000189409	1.6054	0.0469
DKKL1	dickkopf like acrosomal protein 1	ENSG00000104901	1.5993	0.0268
PTGS2	prostaglandin-endoperoxide synthase 2	ENSG00000073756	1.5968	0.0465
AC097468.3	-	ENSG00000272644	1.5732	0.0304
CHKB-DT	CHKB divergent transcript	ENSG00000205559	1.5622	0.0249
PHF24	PHD finger protein 24	ENSG00000122733	1.5550	0.0395
SULT1A2	sulfotransferase family 1A member 2	ENSG00000197165	1.5529	0.0073
ADGRG5	adhesion G protein-coupled receptor G5	ENSG00000159618	1.5427	0.0004

PCSK9	proprotein convertase subtilisin/kexin type 9	ENSG00000169174	1.5334	0.0495
ITGA8	integrin subunit alpha 8	ENSG00000077943	1.5194	0.0236
MESP1	mesoderm posterior bHLH transcription factor 1	ENSG00000166823	1.5055	0.0419
**Down- regulated genes**				
TCAF2C	-	ENSG00000283528	-32.2179	0.0056
HSPB3	heat shock protein family B (small) member 3	ENSG00000169271	-17.7291	0.0139
AL732292.2	-	ENSG00000273416	-17.4308	0.0071
AL365475.1	-	ENSG00000261208	-15.7675	0.0003
AC026740.3	-	ENSG00000286094	-10.3148	0.0011
HAR1B	highly accelerated region 1B	ENSG00000231133	-10.0735	0.0066
LINC01797	-	ENSG00000237179	-9.9344	0.0146
CYP4A11	cytochrome P450 family 4 subfamily A member 11	ENSG00000187048	-9.7878	0.0308
CRHR2	corticotropin releasing hormone receptor 2	ENSG00000106113	-9.2399	0.0053
AC007849.1	-	ENSG00000242795	-7.8240	0.0111
AL162741.1	-	ENSG00000260179	-7.5134	0.0340
AC027601.5	-	ENSG00000279187	-7.3344	0.0390
INE2	-	ENSG00000281371	-7.2866	0.0461
RBFOX3	RNA binding fox-1 homolog 3	ENSG00000167281	-7.0444	0.0034
SERTM2	serine rich and transmembrane domain containing 2	ENSG00000260802	-7.0425	0.0308
IGHV3-43	immunoglobulin heavy variable 3-43	ENSG00000232216	-6.9600	0.0040
MCHR1	melanin concentrating hormone receptor 1	ENSG00000128285	-6.8448	0.0166
DES	desmin	ENSG00000175084	-6.0012	0.0000
ACTG2	actin, gamma 2, smooth muscle, enteric	ENSG00000163017	-5.8912	0.0000
LCMT1-AS2	-	ENSG00000260034	-5.8442	0.0340
ZNF663P	zinc finger protein 663, pseudogene	ENSG00000215452	-5.6175	0.0052
OR4D1	olfactory receptor family 4 subfamily D member 1	ENSG00000141194	-5.6041	0.0288
AL132655.2	novel transcript	ENSG00000268649	-5.4190	0.0201
GLB1L3	galactosidase beta 1 like 3	ENSG00000166105	-5.3165	0.0419
LEFTY2	left-right determination factor 2	ENSG00000143768	-5.1569	0.0002
AC108448.2	-	ENSG00000236710	-5.1111	0.0247
GCKR	glucokinase regulator	ENSG00000084734	-4.8905	0.0163
IGHV4-4	-	ENSG00000276775	-4.8368	0.0306
ZSWIM8-AS1	ZSWIM8 antisense RNA 1	ENSG00000272589	-4.8175	0.0468
AC006042.3	-	ENSG00000233264	-4.5973	0.0481
AC008498.1	-	ENSG00000276945	-4.5361	0.0340
IGHG3	immunoglobulin heavy constant gamma 3 (G3m marker)	ENSG00000211897	-4.3554	0.0182
PGM5-AS1	PGM5 antisense RNA 1	ENSG00000224958	-4.2846	0.0178
ADAMTS9-AS1	ADAMTS9 antisense RNA 1	ENSG00000241158	-4.2108	0.0182
AC000120.2	-	ENSG00000285772	-4.1982	0.0243
AC111152.2	-	ENSG00000259647	-4.1950	0.0438
AC018845.3	-	ENSG00000261173	-4.1629	0.0253
ATXN2-AS	-	ENSG00000258099	-4.1460	0.0142
BX664615.1	-	ENSG00000229273	-4.1279	0.0022
GREM1	gremlin 1, DAN family BMP antagonist	ENSG00000166923	-4.1039	0.0020

AC007744.1	-	ENSG00000271894	-4.0853	0.0473
AL442125.2	-	ENSG00000276916	-4.0497	0.0196
TPTE2P6	transmembrane phosphoinositide 3-phosphatase and tensin homolog 2 pseudogene 6	ENSG00000205822	-3.9845	0.0420
TCF23	transcription factor 23	ENSG00000163792	-3.7790	0.0051
JPH2	junctophilin 2	ENSG00000149596	-3.7386	0.0194
AC093510.2	-	ENSG00000260871	-3.7358	0.0399
SFRP5	secreted frizzled related protein 5	ENSG00000120057	-3.7216	0.0138
IGKV1OR-2	immunoglobulin kappa variable 1/OR-2 (pseudogene)	ENSG00000156755	-3.6770	0.0129
AC092653.1	-	ENSG00000273245	-3.6401	0.0366
MS4A1	membrane spanning 4-domains A1	ENSG00000156738	-3.6084	0.0147
SCN7A	sodium voltage-gated channel alpha subunit 7	ENSG00000136546	-3.6076	0.0371
AP002812.5	-	ENSG00000255449	-3.5911	0.0477
SORCS3	sortilin related VPS10 domain containing receptor 3	ENSG00000156395	-3.5611	0.0163
WSCD2	WSC domain containing 2	ENSG00000075035	-3.4796	0.0073
MPPED1	metallophosphoesterase domain containing 1	ENSG00000186732	-3.3978	0.0196
AC084018.1	-	ENSG00000272849	-3.3392	0.0187
KCNMB1	potassium calcium-activated channel subfamily M regulatory beta subunit 1	ENSG00000145936	-3.2896	0.0180
AL133338.2	-	ENSG00000270987	-3.2496	0.0235
RRH	retinal pigment epithelium-derived rhodopsin homolog	ENSG00000180245	-3.2354	0.0465
CNN1	calponin 1	ENSG00000130176	-3.2212	0.0039
RYR3	ryanodine receptor 3	ENSG00000198838	-3.1946	0.0310
COX6A2	cytochrome c oxidase subunit 6A2	ENSG00000156885	-3.1561	0.0161
SSTR3	-	ENSG00000278195	-3.1420	0.0151
ZNF534	zinc finger protein 534	ENSG00000198633	-3.1096	0.0396
IL12A-AS1	IL12A antisense RNA 1	ENSG00000244040	-3.0590	0.0472
LINC01869	-	ENSG00000180279	-3.0172	0.0382
SLC8A2	solute carrier family 8 member A2	ENSG00000118160	-2.9845	0.0247
GJA3	gap junction protein alpha 3	ENSG00000121743	-2.9793	0.0032
ZNF197-AS1	ZNF197 antisense RNA 1	ENSG00000233509	-2.9641	0.0413
AF287957.1	-	ENSG00000271743	-2.9481	0.0065
ACTC1	actin, alpha, cardiac muscle 1	ENSG00000159251	-2.9185	0.0379
PLN	phospholamban	ENSG00000198523	-2.8969	0.0386
WNT10B	Wnt family member 10B	ENSG00000169884	-2.8959	0.0020
AC007161.3	-	ENSG00000283549	-2.8845	0.0166
PTGIS	prostaglandin I2 synthase	ENSG00000124212	-2.8666	0.0050
PTH2R	parathyroid hormone 2 receptor	ENSG00000144407	-2.8543	0.0076
SNTG2	syntrophin gamma 2	ENSG00000172554	-2.8433	0.0153
NEURL1	neuralized E3 ubiquitin protein ligase 1	ENSG00000107954	-2.8361	0.0038
VAT1L	vesicle amine transport 1 like	ENSG00000171724	-2.8304	0.0154
AP001107.5	-	ENSG00000254510	-2.8263	0.0121
AC119396.1	-	ENSG00000263264	-2.7972	0.0193
IL17B	interleukin 17B	ENSG00000127743	-2.7715	0.0498
ADARB2	adenosine deaminase, RNA specific B2 (inactive)	ENSG00000185736	-2.7674	0.0220
AL121749.1	-	ENSG00000273312	-2.7554	0.0110
AL359510.2	-	ENSG00000280157	-2.7267	0.0064

AP000892.3	-	ENSG00000280143	-2.7171	0.0134
AL353719.1	-	ENSG00000260475	-2.6939	0.0412
HAVCR1	hepatitis A virus cellular receptor 1	ENSG00000113249	-2.6858	0.0203
PTGER3	prostaglandin E receptor 3	ENSG00000050628	-2.6642	0.0099
KCNB1	potassium voltage-gated channel subfamily B member 1	ENSG00000158445	-2.6459	0.0074
ITGB1BP2	integrin subunit beta 1 binding protein 2	ENSG00000147166	-2.6359	0.0272
GAL3ST3	galactose-3-O-sulfotransferase 3	ENSG00000175229	-2.6180	0.0251
KCNMB2	potassium calcium-activated channel subfamily M regulatory beta subunit 2	ENSG00000197584	-2.6126	0.0117
CD36	CD36 molecule	ENSG00000135218	-2.5829	0.0310
LILRP2	leukocyte immunoglobulin-like receptor pseudogene 2	ENSG00000170858	-2.5479	0.0024
MSTN	myostatin	ENSG00000138379	-2.5346	0.0202
FGF17	fibroblast growth factor 17	ENSG00000158815	-2.5209	0.0138
KERA	keratocan	ENSG00000139330	-2.5204	0.0155
AC139491.2	-	ENSG00000248596	-2.5119	0.0116
AC104695.3	-	ENSG00000270210	-2.4957	0.0421
AC093827.3	-	ENSG00000251411	-2.4937	0.0470
ACSM1	acyl-CoA synthetase medium chain family member 1	ENSG00000166743	-2.4647	0.0368
CD79A	CD79a molecule	ENSG00000105369	-2.4497	0.0051
PDLIM3	PDZ and LIM domain 3	ENSG00000154553	-2.4366	0.0255
SLX1A	SLX1 homolog A, structure-specific endonuclease subunit	ENSG00000132207	-2.4298	0.0376
AP003392.5	-	ENSG00000271751	-2.4268	0.0446
PRELP	proline and arginine rich end leucine rich repeat protein	ENSG00000188783	-2.3832	0.0451
SEL1L2	SEL1L2, ERAD E3 ligase adaptor subunit	ENSG00000101251	-2.3824	0.0297
LMOD1	leiomodin 1	ENSG00000163431	-2.3572	0.0042
CHRDL2	chordin like 2	ENSG00000054938	-2.3533	0.0003
AC006116.11	-	ENSG00000285996	-2.3431	0.0231
RERG	RAS like estrogen regulated growth inhibitor	ENSG00000134533	-2.3348	0.0019
AL391845.2	-	ENSG00000233542	-2.3269	0.0383
AC011472.4	-	ENSG00000273733	-2.3266	0.0111
ANGPTL1	angiopoietin like 1	ENSG00000116194	-2.3090	0.0250
CCDC168	coiled-coil domain containing 168	ENSG00000175820	-2.2713	0.0267
LINC01305	-	ENSG00000231453	-2.2552	0.0471
AL391244.1	-	ENSG00000225905	-2.2509	0.0310
AC090772.3	-	ENSG00000265750	-2.2427	0.0148
TAGLN	transgelin	ENSG00000149591	-2.2364	0.0102
SPATA1	spermatogenesis associated 1	ENSG00000122432	-2.2254	0.0194
GUSBP4	glucuronidase, beta pseudogene 4	ENSG00000239650	-2.2165	0.0475
BTNL9	butyrophilin like 9	ENSG00000165810	-2.2000	0.0040
INMT	indolethylamine N-methyltransferase	ENSG00000241644	-2.1987	0.0275
LINC02495	-	ENSG00000249896	-2.1947	0.0040
SMIM38	-	ENSG00000284713	-2.1936	0.0480
LSMEM1	leucine rich single-pass membrane protein 1	ENSG00000181016	-2.1890	0.0263
AC011498.2	-	ENSG00000267030	-2.1666	0.0037
LINC01607	-	ENSG00000272138	-2.1573	0.0148
AC092376.1	-	ENSG00000261722	-2.1473	0.0436

HERC2P8	hect domain and RLD 2 pseudogene 8	ENSG00000261599	-2.1460	0.0388
LINC01936	-	ENSG00000235997	-2.1439	0.0094
AC127521.1	-	ENSG00000262823	-2.1189	0.0329
AL360181.2	-	ENSG00000235245	-2.1038	0.0261
KCNQ4	potassium voltage-gated channel subfamily Q member 4	ENSG00000117013	-2.0969	0.0307
SLC2A4	solute carrier family 2 member 4	ENSG00000181856	-2.0896	0.0357
GPC3	glypican 3	ENSG00000147257	-2.0701	0.0145
AC108925.1	-	ENSG00000286010	-2.0691	0.0259
PPM1N	protein phosphatase, Mg2+/Mn2+ dependent 1N (putative)	ENSG00000213889	-2.0625	0.0405
Z93930.2	-	ENSG00000226471	-2.0624	0.0376
SP9	Sp9 transcription factor	ENSG00000217236	-2.0588	0.0186
OR2C3	olfactory receptor family 2 subfamily C member 3	ENSG00000196242	-2.0352	0.0302
ACTG1P17	-	ENSG00000259315	-2.0335	0.0188
CRB2	crumbs cell polarity complex component 2	ENSG00000148204	-2.0294	0.0193
LINGO2	leucine rich repeat and Ig domain containing 2	ENSG00000174482	-2.0262	0.0110
PCDHA7	protocadherin alpha 7	ENSG00000204963	-1.9913	0.0102
PCP2	Purkinje cell protein 2	ENSG00000174788	-1.9543	0.0245
REP15	RAB15 effector protein	ENSG00000174236	-1.9366	0.0043
ITGA10	integrin subunit alpha 10	ENSG00000143127	-1.9358	0.0228
ZCCHC12	zinc finger CCHC-type containing 12	ENSG00000174460	-1.9244	0.0035
LINC01366	-	ENSG00000235172	-1.9146	0.0348
SORCS1	sortilin related VPS10 domain containing receptor 1	ENSG00000108018	-1.9130	0.0140
AC116407.2	-	ENSG00000277511	-1.9118	0.0412
GPR55	G protein-coupled receptor 55	ENSG00000135898	-1.9076	0.0271
PCDHGB5	-	ENSG00000276547	-1.8931	0.0041
PPP1R12B	protein phosphatase 1 regulatory subunit 12B	ENSG00000077157	-1.8808	0.0041
ACTA2	actin, alpha 2, smooth muscle, aorta	ENSG00000107796	-1.8762	0.0264
PLXNB3	plexin B3	ENSG00000198753	-1.8701	0.0149
AKR1C2	aldo-keto reductase family 1 member C2	ENSG00000151632	-1.8660	0.0375
MAMDC2	MAM domain containing 2	ENSG00000165072	-1.8482	0.0035
CACNB2	calcium voltage-gated channel auxiliary subunit beta 2	ENSG00000165995	-1.8458	0.0278
AKR7L	aldo-keto reductase family 7 like (gene/pseudogene)	ENSG00000211454	-1.8362	0.0256
AL662899.2	novel protein	ENSG00000263020	-1.8360	0.0110
AC114490.1	-	ENSG00000241014	-1.8338	0.0070
AC109449.1	-	ENSG00000259940	-1.8313	0.0408
AC026202.2	-	ENSG00000233912	-1.8113	0.0221
PGM5	phosphoglucomutase 5	ENSG00000154330	-1.8100	0.0492
CCDC144NL-AS1	-	ENSG00000233098	-1.7998	0.0114
SLC7A3	solute carrier family 7 member 3	ENSG00000165349	-1.7964	0.0371
AL355312.2	-	ENSG00000231760	-1.7901	0.0300
AC126773.2	-	ENSG00000260577	-1.7788	0.0282
FBXL22	F-box and leucine rich repeat protein 22	ENSG00000197361	-1.7728	0.0065
TRPC4	transient receptor potential cation channel subfamily C member 4	ENSG00000133107	-1.7699	0.0374

GAS1	growth arrest specific 1	ENSG00000180447	-1.7604	0.0026
HIST2H2BA	histone cluster 2 H2B family member a (pseudogene)	ENSG00000223345	-1.7595	0.0173
AC092667.1	-	ENSG00000230393	-1.7474	0.0355
NFASC	neurofascin	ENSG00000163531	-1.7422	0.0404
RSPO3	R-spondin 3	ENSG00000146374	-1.7355	0.0187
LINC00622	long intergenic non-protein coding RNA 622	ENSG00000260941	-1.7348	0.0411
RIMS1	regulating synaptic membrane exocytosis 1	ENSG00000079841	-1.7343	0.0385
RASSF10	Ras association domain family member 10	ENSG00000189431	-1.7305	0.0351
PIPOX	pipecolic acid and sarcosine oxidase	ENSG00000179761	-1.7198	0.0316
SORBS1	sorbin and SH3 domain containing 1	ENSG00000095637	-1.7106	0.0243
HIF3A	hypoxia inducible factor 3 subunit alpha	ENSG00000124440	-1.7035	0.0464
ASB2	ankyrin repeat and SOCS box containing 2	ENSG00000100628	-1.6956	0.0190
AC133552.2	-	ENSG00000262587	-1.6917	0.0450
TMEM145	transmembrane protein 145	ENSG00000167619	-1.6872	0.0284
NCAM1	neural cell adhesion molecule 1	ENSG00000149294	-1.6858	0.0274
SLC25A27	solute carrier family 25 member 27	ENSG00000153291	-1.6850	0.0025
CAHM	colon adenocarcinoma hypermethylated	ENSG00000270419	-1.6812	0.0391
PCDHGA1	protocadherin gamma subfamily A, 1	ENSG00000204956	-1.6753	0.0078
AP003774.1	novel transcript	ENSG00000181908	-1.6676	0.0333
AC156455.1	-	ENSG00000256546	-1.6674	0.0166
ANGPT1	angiopoietin 1	ENSG00000154188	-1.6641	0.0136
DRD2	dopamine receptor D2	ENSG00000149295	-1.6503	0.0453
KIR2DL1	killer cell immunoglobulin like receptor, two Ig domains and long cytoplasmic tail 1	ENSG00000125498	-1.6435	0.0111
KIR2DL3	killer cell immunoglobulin like receptor, two Ig domains and long cytoplasmic tail 3	ENSG00000243772	-1.6424	0.0229
AP000873.2	-	ENSG00000247137	-1.6415	0.0280
NEXN	nexilin F-actin binding protein	ENSG00000162614	-1.6395	0.0187
AC103740.1	-	ENSG00000259370	-1.6385	0.0227
AC083843.3	-	ENSG00000259820	-1.6351	0.0115
LINC02610	long intergenic non-protein coding RNA 2610	ENSG00000186235	-1.6342	0.0142
TAGAP	T cell activation RhoGTPase activating protein	ENSG00000164691	-1.6330	0.0095
ALDH1A3	aldehyde dehydrogenase 1 family member A3	ENSG00000184254	-1.6326	0.0052
AC015813.2	novel transcript	ENSG00000266086	-1.6320	0.0389
CARMN	cardiac mesoderm enhancer-associated non-coding RNA	ENSG00000249669	-1.6277	0.0440
TUBA3E	tubulin alpha 3e	ENSG00000152086	-1.6255	0.0382
ISM2	isthmin 2	ENSG00000100593	-1.6182	0.0332
RFLNA	refilin A	ENSG00000178882	-1.6136	0.0421
AC084824.1	-	ENSG00000257511	-1.6020	0.0312
GATA6	GATA binding protein 6	ENSG00000141448	-1.6019	0.0221
PDZRN3-AS1	PDZRN3 antisense RNA 1	ENSG00000239677	-1.5997	0.0120
SHISAL1	shisa like 1	ENSG00000138944	-1.5994	0.0219
SNAP25	synaptosome associated protein 25	ENSG00000132639	-1.5839	0.0398
SPEG	striated muscle enriched protein kinase	ENSG00000072195	-1.5825	0.0430
ZBP1	Z-DNA binding protein 1	ENSG00000124256	-1.5753	0.0243

TPTE2P1	transmembrane phosphoinositide 3-phosphatase and tensin homolog 2 pseudogene 1	ENSG00000253771	-1.5691	0.0058
SCNN1D	sodium channel epithelial 1 delta subunit	ENSG00000162572	-1.5634	0.0052
LINC01355	-	ENSG00000261326	-1.5470	0.0393
AC105345.1	-	ENSG00000205959	-1.5354	0.0133
TSPAN32	tetraspanin 32	ENSG00000064201	-1.5348	0.0343
LIMS2	LIM zinc finger domain containing 2	ENSG00000072163	-1.5325	0.0270
WBP2NL	WBP2 N-terminal like	ENSG00000183066	-1.5262	0.0175
AC002094.1	-	ENSG00000258924	-1.5204	0.0235
CSDC2	cold shock domain containing C2	ENSG00000172346	-1.5192	0.0352
NPIPB14P	-	ENSG00000226232	-1.5164	0.0467
AL355987.4	-	ENSG00000273066	-1.5144	0.0213
WIPF3	WAS/WASL interacting protein family member 3	ENSG00000122574	-1.5122	0.0087
SYNPO2	synaptopodin 2	ENSG00000172403	-1.5103	0.0105
PVRIG	PVR related immunoglobulin domain containing	ENSG00000213413	-1.5092	0.0125
FAM225A	family with sequence similarity 225 member A	ENSG00000231528	-1.5067	0.0042
FILIP1L	filamin A interacting protein 1 like	ENSG00000168386	-1.5036	0.0125
AC092376.2	-	ENSG00000277954	-1.5017	0.0401
MYL9	myosin light chain 9	ENSG00000101335	-1.5013	0.0181

*P/C: polyp/control

Table S3. Comparison of expression levels of genes related to the Wnt signal pathway and muscle function.

Symbol	Gene description	Gene ID	Fold change - P/C*	p-Value
**Wnt signaling pathway-related genes**				
DKK1	dickkopf WNT signaling pathway inhibitor 1	ENSG00000107984	2.4271	0.0239
DKKL1	dickkopf like acrosomal protein 1	ENSG00000104901	1.5993	0.0268
WNT10B	Wnt family member 10B	ENSG00000169884	-2.8959	0.0020
GREM1	gremlin 1, DAN family BMP antagonist	ENSG00000166923	-4.1039	0.0020
RSPO3	R-spondin 3	ENSG00000146374	-1.7355	0.0187
SFRP5	secreted frizzled related protein 5	ENSG00000120057	-3.7216	0.0138
GPC3	glypican 3	ENSG00000147257	-2.0701	0.0145
**Muscle function-related genes**				
BUB1B-PAK6	BUB1B-PAK6 readthrough	ENSG00000259288	1.9489	0.0287
ITGA8	integrin subunit alpha 8	ENSG00000077943	1.5194	0.0236
COX6A2	cytochrome c oxidase subunit 6A2	ENSG00000156885	-3.1561	0.0161
TUBA3E	tubulin alpha 3e	ENSG00000152086	-1.6255	0.0382
KCNMB2	potassium calcium-activated channel subfamily M regulatory beta subunit 2	ENSG00000197584	-2.6126	0.0117
ITGA10	integrin subunit alpha 10	ENSG00000143127	-1.9358	0.0228
ACTC1	actin, alpha, cardiac muscle 1	ENSG00000159251	-2.9185	0.0379
MYL9	myosin light chain 9	ENSG00000101335	-1.5013	0.0181
ACTA2	actin, alpha 2, smooth muscle, aorta	ENSG00000107796	-1.8762	0.0264
PPP1R12B	protein phosphatase 1 regulatory subunit 12B	ENSG00000077157	-1.8808	0.0041
DES	desmin	ENSG00000175084	-6.0012	1.44×10^{-9}
PLN	phospholamban	ENSG00000198523	-2.8969	0.0386
SLC8A2	solute carrier family 8 member A2	ENSG00000118160	-2.9845	0.0247
KCNMB1	potassium calcium-activated channel subfamily M regulatory beta subunit 1	ENSG00000145936	-3.2896	0.0180

*P/C: polyp/control

Table S4. Summary of real-time PCR Primers.

Gene name	Accession no.	Forward primers (5′ to 3′)	Reverse primers (5′ to 3′)	Product size (bp)
ACTG2	NM_001615	GACAGGATGCAGAAGGAGA	TGGAAGGTGGAGAGAGAGG	128
CNN1	NM_001299	TTCCCTGTTTTCCCCCCA	CTCTCTCCAAACTCTAACCCT	137
DES	NM_001382709	GCTGCTGGACTTCTCACT	GCGCACCTTCTCGATGTA	121
GPC3	NM_004484	ACCATCAAGTTGTGCCAAA	GGAGGGAGTGAGAGAGAAAG	80
HOXA13	NM_000522	GGGAGAGAAAAGAAAGGAGGAA	GACACATGCAGACCCAAC	118
LEFTY2	NM_003240	TTCCCCCAAATACTGACCC	GCCCTTCATCCTTCCTCT	90
LMOD1	NM_012134	GAAAAAGGAGGATGAGAAGGT	GGTGTTTTGGTCTTGCTG	129
PTGIS	NM_000961	TCCTCCTCTTCCCCTTCCT	GCCGTTTCCCATCCTTGT	132
RERG	NM_032918	TTCCCCTTCTGTTCCCCT	CACGCTAAAACTTCCCAACC	120
TAGLN	NM_003186	CCAGACTGTTGACCTCTT	CTCCTGCGCTTTCTTCAT	139
PUM1	NM_001020658	CACCTCCTTCCCTCTCTT	ATGTATCTTCCACTGCCATT	79
PRDM4	NM_012406	CTAACCAAATACCATCTCACCC	TCATCCTCTTCTTCCTCCTCT	89
YWHAZ	NM_001135699	GCTCTGGCTTATGGTCTTT	TCTTTCTCTTTCTCCCCCC	82
